# Characterizing Technology Use and Preferences for Health Communication in South Asian Immigrants With Prediabetes or Diabetes: Cross-Sectional Descriptive Study

**DOI:** 10.2196/52687

**Published:** 2024-04-26

**Authors:** Lu Hu, Laura C Wyatt, Farhan Mohsin, Sahnah Lim, Jennifer Zanowiak, Shinu Mammen, Sarah Hussain, Shahmir H Ali, Deborah Onakomaiya, Hayley M Belli, Angela Aifah, Nadia S Islam

**Affiliations:** 1 Department of Population Health Center for Healthful Behavior Change, Institute for Excellence in Health Equity New York University Grossman School of Medicine New York, NY United States; 2 Department of Population Health New York University Grossman School of Medicine New York, NY United States; 3 Department of Social and Behavioral Sciences School of Global Public Health New York University New York, NY United States; 4 Vilcek Institute of Graduate Biomedical Sciences New York University Grossman School of Medicine New York, NY United States

**Keywords:** South Asian immigrants, type 2 diabetes, technology access, technology use, prediabetes, health disparities, mHealth, health equity, immigrant health, mobile health, smartphone, diabetes, diabetic, DM, diabetes mellitus, immigrants, prevention, regression, regression model, logistic regression, mobile health interventions

## Abstract

**Background:**

Type 2 diabetes disproportionately affects South Asian subgroups. Lifestyle prevention programs help prevent and manage diabetes; however, there is a need to tailor these programs for mobile health (mHealth).

**Objective:**

This study examined technology access, current use, and preferences for health communication among South Asian immigrants diagnosed with or at risk for diabetes, overall and by sex. We examined factors associated with interest in receiving diabetes information by (1) text message, (2) online (videos, voice notes, online forums), and (3) none or skipped, adjusting for sociodemographic characteristics and technology access.

**Methods:**

We used baseline data collected in 2019-2021 from two clinical trials among South Asian immigrants in New York City (NYC), with one trial focused on diabetes prevention and the other focused on diabetes management. Descriptive statistics were used to examine overall and sex-stratified impacts of sociodemographics on technology use. Overall logistic regression was used to examine the preference for diabetes information by text message, online (videos, voice notes, or forums), and no interest/skipped response.

**Results:**

The overall sample (N=816) had a mean age of 51.8 years (SD 11.0), and was mostly female (462/816, 56.6%), married (756/816, 92.6%), with below high school education (476/816, 58.3%) and limited English proficiency (731/816, 89.6%). Most participants had a smartphone (611/816, 74.9%) and reported interest in receiving diabetes information via text message (609/816, 74.6%). Compared to male participants, female participants were significantly less likely to own smartphones (317/462, 68.6% vs 294/354, 83.1%) or use social media apps (Viber: 102/462, 22.1% vs 111/354, 31.4%; WhatsApp: 279/462, 60.4% vs 255/354, 72.0%; Facebook: Messenger 72/462, 15.6% vs 150/354, 42.4%). A preference for receiving diabetes information via text messaging was associated with male sex (adjusted odds ratio [AOR] 1.63, 95% CI 1.01-2.55; *P*=.04), current unemployment (AOR 1.62, 95% CI 1.03-2.53; *P*=.04), above high school education (AOR 2.17, 95% CI 1.41-3.32; *P*<.001), and owning a smart device (AOR 3.35, 95% CI 2.17-5.18; *P*<.001). A preference for videos, voice notes, or online forums was associated with male sex (AOR 2.38, 95% CI 1.59-3.57; *P*<.001) and ownership of a smart device (AOR 5.19, 95% CI 2.83-9.51; *P*<.001). No interest/skipping the question was associated with female sex (AOR 2.66, 95% CI 1.55-4.56; *P*<.001), high school education or below (AOR 2.02, 95% CI 1.22-3.36; *P*=.01), not being married (AOR 2.26, 95% CI 1.13-4.52; *P*=.02), current employment (AOR 1.96, 95% CI 1.18-3.29; *P*=.01), and not owning a smart device (AOR 2.06, 95% CI 2.06-5.44; *P*<.001).

**Conclusions:**

Technology access and social media usage were moderately high in primarily low-income South Asian immigrants in NYC with prediabetes or diabetes. Sex, education, marital status, and employment were associated with interest in mHealth interventions. Additional support to South Asian women may be required when designing and developing mHealth interventions.

**Trial Registration:**

ClinicalTrials.gov NCT03333044; https://classic.clinicaltrials.gov/ct2/show/NCT03333044, ClinicalTrials.gov NCT03188094; https://classic.clinicaltrials.gov/ct2/show/NCT03188094

**International Registered Report Identifier (IRRID):**

RR2-10.1186/s13063-019-3711-y

## Introduction

The South Asian population represents one of the fastest-growing minoritized groups in the United States [1]. Between 2010 and 2017, the population size grew from 3.5 million to 5.4 million, representing an increase of 40% [2]. South Asians include a diverse group of individuals with ancestry from India, Bangladesh, Pakistan, Nepal, Bhutan, Sri Lanka, and the Maldives, and the majority of South Asian individuals in the United States are foreign-born immigrants [1,2]. Approximately 10% of South Asian immigrants in the United States live in poverty, with immigrants from Bhutan (33.3%), Bangladesh (24.2%), Nepal (23.9%), and Pakistan (15.8%) having the highest poverty rates [3]. In New York City (NYC), compared to the 13% poverty rate among the non-Hispanic White population, poverty rates were reported to be much higher for the Bangladeshi (32%) and Pakistani (29%) populations [4]. A significant proportion of South Asian individuals have limited English proficiency (LEP) and are engaged in low-wage jobs (eg, taxi drivers, cashiers, and restaurant workers) [3].

Type 2 diabetes (T2D) disproportionately affects many racial/ethnic minorities in the United States, including the South Asian population [5]. The MASALA study, conducted between 2010 and 2013, found that the age-adjusted prevalence of T2D was 23% among individuals of South Asian ethnicity, compared to a prevalence of 9.4%, 13.3%, and 10.3% in the White, African American, and Hispanic American populations, respectively [5,6]. Similar patterns were observed in another population-based study, which reported that T2D prevalence was 17% in US Asian Indian, 15% in Native American/Alaskan native, 13% in non-Hispanic Black, 10% in Hispanic, and 8% in non-Hispanic White populations [7]. Given the high economic and societal costs of T2D, there is an urgent need to develop interventions to prevent and manage the T2D burden in the South Asian population.

Lifestyle counseling programs such as the National Diabetes Prevention Program or Diabetes Self-Management Education and Support program are evidence-based interventions that provide important counseling and support to individuals to manage or prevent T2D [8,9]. However, access to these evidence-based programs has been significantly limited among underserved immigrant communities [10]. First, these programs are often offered during in-person visits, which can be a significant barrier for many low-income South Asian immigrants who do not have a driver’s license or do not know how to navigate the public transportation system [11,12]. Second, most of these existing programs lack cultural and linguistic tailoring of the content [13]. A significant percentage of South Asian immigrants reported LEP and had difficulty understanding their health care providers during office visits [11,13]. In addition, the lack of cultural tailoring can make it challenging for this group of immigrants to initiate dietary and behavioral changes. Lastly, a lack of insurance or other financial barriers can further prevent South Asian immigrants from benefitting from these programs [14].

Given such barriers, mobile health (mHealth) has been rapidly growing over the past few decades and could be a promising approach to increase access to these evidence-based programs among South Asian immigrants [15,16]. Several studies suggest that text message–based interventions among minoritized populations can improve glycemic control for patients with T2D [17-19]. Using text messaging and social media platforms may be promising for immigrant populations, owing to their social needs to stay connected with friends and families in their home countries [20-22]. One mHealth study conducted in India found that participants receiving motivational messages to reduce diabetes risk behaviors had a greater improvement in health behaviors when compared to that of control participants [23].

The inclusion of culturally tailored mHealth interventions among South Asian individuals living in the United States has been largely limited [24,25]. While several research teams have reported using telephones to follow up or conduct motivational interviews with participants [26-28], the use of smartphone or remote technologies remains understudied among South Asian immigrants and there is little data characterizing the use and access of mHealth tools in this population. In a post–COVID-19 world, such information is urgently needed, given the increasing interest in telehealth. To address this knowledge gap, this study leveraged baseline data from the intervention groups of two parent clinical trials whose primary aims were to examine the effectiveness of a community-clinical linkage intervention on diabetes prevention and control in a primarily low-income South Asian immigrant population.

For this study, we examined technology access, current use, and factors associated with preferences for health communication among South Asian immigrants in NYC. Given that South Asian women bear a higher T2D burden [29] than their male counterparts and face significant cultural barriers to health care–seeking behaviors [30], we also examined whether there were sex differences in technology access, current use, and preferences for health communication. Finally, we examined factors associated with interest in receiving diabetes information by (1) text message, (2) online (videos, voice notes, online forums), and (3) none or skipped, adjusting for sociodemographics and technology access. This information will serve as a critical first step to the development of tailored mHealth interventions for the South Asian immigrant population in the United States.

## Methods

### Study Design

We used data collected in two ongoing clinical trials focused on diabetes prevention and management among South Asian immigrants living in NYC (ClinicalTrials.gov NCT03333044 and NCT03188094) [31,32]. Both trials aimed to examine the effectiveness of an integrated community-clinical linkage intervention on health outcomes among low-income South Asian immigrants. For the diabetes prevention trial, the primary outcome was the proportion of participants achieving 5% weight loss at the 6-month follow-up. For the diabetes management trial, the primary outcome was the proportion of participants achieving a hemoglobin A_1c_ (HbA_1c_) value of 7% or less at the 6-month follow-up. For both trials, primary data outcomes included measurements taken from electronic health records and survey data were only collected from intervention group participants; control group participants were not contacted. We conducted a cross-sectional analysis of deidentified data collected during the baseline period of the intervention for the participants from these two parent studies.

### Sample Recruitment

Seven bilingual community health workers (CHWs) led the recruitment effort of the parent clinical trials. Participants were recruited from 18 primary care practice sites in NYC serving primarily South Asian patients (>70%). Registry reports were generated at each site to identify potentially eligible patients. Following the randomization of potential participants on the registry list, an introduction letter was mailed to all patients randomized to the treatment group. After 1-2 weeks, a CHW followed up with a phone call to provide further information.

To be eligible for the parent study, participants had to: (1) self-identify as South Asian; (2) be between 21-75 years old; (3) have an appointment with a physician for routine nonemergency primary care in the last 12 months; (4) have a diagnosis of diabetes for at least 12 months (for diabetes study participants); (5) have an HbA_1c_ of at least 7% in the last 12 months (for diabetes study participants); and (6) have a BMI of ≥23 kg/m^2^, as the threshold for overweight for Asian individuals [33] (for prediabetes study participants).

The study team first finalized all materials (flyers, consent forms, surveys) in English. One of the bilingual CHWs performed the translation and then conducted back-translation to account for discrepancies. Translated documents were then reviewed by separate bilingual CHWs to ensure accuracy and use of lay language. If there was any discrepancy, the group of CHWs met with the administrative team to reach an agreement. All study materials were available in English, Bengali, Urdu, and Punjabi. Surveys were administered by bilingual CHWs in the participant’s preferred language via face-to-face interviews or over the phone between 2019 and 2021.

### Ethical Considerations

The parent studies (S17-00693 and S17-01479) were approved by the New York University Grossman School of Medicine Institutional Review Board (IRB). All participants provided informed consent. The parent IRB approvals cover the secondary analysis of deidentified data without additional consent from participants. In the parent studies, participants received a US $20 gift card for completion of the 6-month follow-up survey. The data used in the analyses were deidentified.

### Outcome Variables

The outcome of interest was the participants’ preference in receiving diabetes information through the following modalities: (1) text message, (2) online (videos, voice notes, online forum), or (3) none/skipped question (suggesting no interest).

### Predictor Variables

Sociodemographic information included age (continuous), sex, education level (high school or below vs above high school), marital status (married vs not married), employment status (employed, not employed, retired), LEP (speaking English less than very well vs well, not well, or not at all), and number of years lived in the United States (continuous).

Questions were adapted from the National Cancer Institute Health Information National Trends Survey [34] to assess technology access, current use of technology for health management, current social media use, and interest in mHealth interventions. The questions were reviewed by bilingual CHWs and our community advisory board for cultural relevance. Participants were asked if they owned a basic mobile phone, a smartphone, a tablet, or none of these (all options that apply could be selected). Participants owning a smartphone or tablet were asked whether any health-related apps were installed on the device (“yes” vs “no” or “don’t know”). All participants were asked if Wi-Fi was installed at home (“yes” vs “no”) and if they had used an electronic device (eg, Fitbit, blood glucose meter, or blood pressure monitor) to track their health in the past 12 months. Participants were asked if, over the past 30 days, they had used: (1) Viber, (2) WhatsApp, (3) basic text messaging through a phone carrier, (4) iMessage, (5) Facebook Messenger, and (6) IMO (a messaging app that is popular in South Asian countries).

### Statistical Analyses

Descriptive statistics were used to examine the distribution of sociodemographic variables, technology access, current use, and interest in receiving diabetes information in the future in the overall sample and stratified by sex. Means and SDs are reported for continuous variables, whereas frequencies and percentages are reported for categorical variables. Differences by sex were assessed using *t* tests and *χ*^2^ tests.

Bivariate analyses were run to examine each outcome and all potential predictor variables. The *χ*^2^ test was used for comparisons of categorical variables and ANOVA was used for comparisons of continuous variables. All variables found to be significant at *P*<.01 (with any of the three outcomes) were included in the initial regression models, along with variables that were not statistically significant in bivariate analyses but with theoretical significance. A total of three multivariable logistic regression models were used to examine factors associated with interest in receiving diabetes information via different mHealth modalities (dependent variable), including (1) by text message, (2) via combined online format (videos, voice notes, or online forum), and (3) not interested in receiving any diabetes information or skipped the question. In these multivariable logistic regression models, independent variables included age, sex, education, marital status, employment status, years in the United States, English proficiency, access to Wi-Fi at home, and access to a smart device. Adjusted odds ratios and 95% CIs were calculated. All analyses were performed with SPSS version 28.

## Results

### Participant Characteristics

The sample included 816 participants, with 413 (50.6%) in the prediabetes study and 403 (49.4%) in the diabetes study. The mean age of the participants was 51.8 (SD 11.0, range 22-75) years; 92.6% (756/816) of the participants were married and 89.6% (731/816) reported LEP. More than half the sample identified as female (462/816, 56.6%), reported a high school education or less (476/816, 58.3%), and were unemployed or retired (431/816, 52.8%). The majority of individuals were Bangladeshi, followed by Indian, Pakistani, Indo-Caribbean, and Nepali. All were born outside of the United States and the mean time lived in the United States was 12.9 (SD 8.7) years (Table 1).

There were sex differences in most sociodemographic characteristics (Table 1). Compared to male participants, female participants were significantly more likely to be younger, living in the United States for less time, have a high school education or below, be unemployed or retired (334/462, 72.3%; 97/354, 27.4%), and report LEP (Table 1).

**Table 1:** Participant characteristics.

Variables			Total sample (N=816)		Female (n=462)		Male (n=354)		*P* value^a^
**Age (years)**									.03
	Mean (SD)	51.8 (11.0)		51.0 (10.2)		52.7 (12.0)			
	Median (range)	23 (22-75)		52 (25-74)		54 (22-75)			
**Marital status, n (%)**									.01
	Married	756 (92.6)		419 (90.7)		337 (95.2)			
	Not married	54 (6.6)		39 (8.4)		15 (4.2)			
	Skipped	6 (0.7)		4 (0.9)		2 (0.6)			
**Highest education level, n (%)**									<.001
	≤High school	476 (58.3)		329 (71.2)		147 (41.5)			
	>High school	334 (40.9)		129 (27.9)		205 (57.9)			
	Skipped	6 (0.7)		4 (0.9)		2 (0.6)			
**Employment status, n (%)**									<.001
	Currently employed	381 (46.7)		125 (27.1)		256 (72.3)			
	Not employed, not working	381 (46.7)		324 (70.1)		57 (16.1)			
	Retired	50 (6.1)		10 (2.2)		40 (11.3)			
	Skipped	4 (0.5)		3 (0.6)		1 (0.3)			
**Ethnicity**									<.001
	Bangladeshi	522 (64.0)		283 (61.3)		239 (67.5)			
	Indian	119 (14.6)		67 (14.5)		52 (14.7)			
	Pakistani	87 (10.7)		67 (14.5)		20 (5.6)			
	Nepali	27 (3.3)		8 (1.7)		19 (54)			
	Indo-Caribbean	61 (7.5)		37 (8.0)		24 (6.8)			
**Number of years lived in the United States**									<.001
	Valid responses, n	799		449		350			
	Mean (SD)	12.9 (8.7)		11.6 (7.8)		13.9 (9.8)		<.001	
	Median (range)	11 (0.3-45)		10 (0.3-37)		12 (0.8-45)			
**English proficiency, n (%)**									.03
	Speaks English less than very well (LEP^b^)	731 (89.6)		421 (91.1)		310 (87.6)			
	Speaks English very well	75 (9.2)		33 (7.1)		42 (11.9)			
	Skipped	10 (1.2)		8 (1.7)		2 (0.6)			

^a^*P* values do not include missing responses.

^b^LEP: limited English proficiency.

### Technology Ownership and Current Use

As shown in Table 2, nearly three-quarters of the participants reported having a smartphone, whereas less than 2% reported having a tablet. Of those with a smart device (smartphone or tablet), 22.1% (136/615) reported having apps related to health or wellness. Approximately one-quarter of the sample used an electronic device or monitor (eg, blood glucose meter, Fitbit band) to track their health in the past 12 months. The majority reported having Wi-Fi at home. The most common social media forms used in the past 30 days included basic text message via a cellular carrier and WhatsApp, followed by Viber and Facebook Messenger. When asked about interest in receiving diabetes information, three-quarters of the participants indicated interest in receiving diabetes-related information via text message, followed by videos, while 17.6% (144/816) of the participants were not interested in receiving diabetes information or skipped the question.

**Table 2:** Technology access, current social media use, and interest in mobile health interventions, stratified by sex.

Variable				Total sample (N=816), n (%)		Female (n=462), n (%)		Male (n=354), n (%)		*P* value^a^	
**Technology access**											
	**Mobile device ownership**										
		Basic mobile phone	144 (17.6)		106 (22.9)		38 (10.7)		<.001		
		Smartphone	611 (74.9)		317 (68.6)		294 (83.1)		<.001		
		Tablet	15 (1.8)		7 (1.5)		8 (2.3)		.45		
		Missing	5 (0.6)		4 (0.9)		1 (0.3)				
	**Has a mobile device (basic mobile phone or smart device)**										.05
		Yes	751 (92.0)		417 (90.3)		334 (94.4)				
		No	60 (7.4)		41 (8.9)		19 (5.4)				
		Skipped	5 (0.6)		4 (0.9)		5 (0.6)				
	**Has a smart device (smartphone or tablet)**										<.001
		Yes	615 (75.4)		319 (69.0)		296 (83.6)				
		No	196 (24.0)		139 (30.1)		57 (16.1)				
		Skipped	5 (0.6)		4 (0.9)		1 (0.3)				
	**Has apps related to health and wellness on smartphones or tablets (among those with smart devices)**										.01
		Yes	136 (22.1)		55 (17.3)		81 (27.3)				
		No or don’t know	443 (72.0)		240 (75.2)		203 (68.6)				
		Skipped	36 (5.9)		24 (7.5)		12 (4.1)				
	**Other than a tablet or smartphone, have you used an electronic device or monitor to track your health within the last 12 months?**										.10
		Yes	211 (25.9)		127 (27.5)		84 (23.7)				
		No or don’t know	531 (65.1)		283 (61.3)		248 (70.1)				
		Skipped	74 (9.1)		52 (11.3)		22 (6.2)				
**Current social media use**											
	**Has Wi-Fi installed at home**										.33
		Yes	647 (79.3)		362 (78.4)		285 (80.5)				
		No or don’t know	98 (12.0)		62 (13.4)		36 (10.2)				
		Skipped	71 (8.7)		38 (8.2)		33 (9.3)				
	**Types of social media used in the past 30 days**										
		Viber	213 (26.1)		102 (22.1)		111 (31.4)		.004		
		Basic text messages via cellular carrier	561 (68.8)		299 (64.7)		262 (74.0)		.01		
		iMessage	114 (14.0)		64 (13.9)		50 (14.1)		>.99		
		WhatsApp	534 (65.4)		279 (60.4)		255 (72.0)		.001		
		Facebook Messenger	222 (27.2)		72 (15.6)		150 (42.4)		<.001		
		IMO	71 (8.7)		31 (6.7)		40 (11.3)		.02		
**Interest in mobile health interventions^b^**											
	Text message (eg, through phone carrier or WhatsApp)		609 (74.6)		323 (69.9)		286 (80.8)		<.001		
	Videos (eg, through WhatsApp or Viber)		225 (27.6)		88 (19.0)		137 (38.7)		<.001		
	Voice notes (eg, through WhatsApp)		45 (5.5)		22 (4.8)		23 (6.5)		.28		
	Online forum or online support group (eg, on Facebook)		24 (2.9)		10 (2.2)		14 (4.0)		.15		
	None/skipped		144 (17.6)		106 (22.9)		38 (10.7)		<.001		

^a^*P* values do not include missing responses.

^b^Assessed by responding to the question “Would you be interested in receiving diabetes information through any of the following ways? (Select all that apply).”

Similar to the sample characteristics, we also noted differences by sex (Table 2). Compared to male participants, female participants were significantly less likely to own a smartphone; have health-related apps on their smart devices; use Viber, basic text messages, WhatsApp, IMO, and Facebook Messenger among those with a smartphone or tablet; or report interest in receiving diabetes intervention via text message or videos. Conversely, female participants were significantly more likely to have a basic mobile phone and to report no interest or skipped the question about receiving diabetes intervention information compared to male participants.

### Factors Associated With Interest in Receiving Diabetes Intervention

Table 3 provides a summary of the regression analysis to identify factors associated with an interest in receiving diabetes information. The significant factors associated with receiving information via text messaging included male sex, current unemployment, above high school education, and owning a smartphone or tablet.

When combining responses on videos, voice notes, and online forums/online support groups, participants who identified as male and who had a smartphone or tablet were more likely to report an interest in receiving diabetes information through theses online formats.

Factors associated with no interest in receiving diabetes information or skipping the question included female sex, not married, currently employed, high school education or below, and not owning a smart device.

**Table 3:** Factors associated with interest in receiving diabetes information by modality based on multivariable regression.

Variables		Text message			Online (videos, voice notes, online forum)			None/skipped	
		AOR^a^ (95% CI)	*P* value	AOR (95% CI)		*P* value	AOR (95% CI)		*P* value
**Sex**									
	Female	Reference	N/A^b^	Reference		N/A	*2.66 (1.55-4.56)*		*<.001*
	Male	*1.63 (1.04-2.55)^c^*	*.04*	*2.38 (1.59-3.57)*		*<.001*	Reference		N/A
Age (years)		1.00 (0.98-1.02)	.66	1.00 (0.98-1.02)		.86	1.00 (0.97-1.02)		.65
**Marital status**									
	Married	1.92 (0.99-3.72)	.05	1.99 (0.91-4.37)		.09	Reference		N/A
	Not married	Reference	N/A	Reference		N/A	*2.26 (1.13-4.52)*		*.02*
**Education**									
	≤High school	Reference	N/A	Reference		N/A	*2.* *02 (1.22-3.36)*		*.01*
	>High school	*2.17 (1.41-3.32)*	*<.001*	1.15 (0.80-1.65)		.47	Reference		N/A
**Employment**									
	Employed	Reference	N/A	Reference		N/A	*1.96 (1.18-3.29)*		*.01*
	Unemployed	*1.62 (1.03-2.53)*	*.04*	1.02 (0.68-1.52)		.94	Reference		N/A
Years in the United States		0.99 (0.97-1.01)	.20	1.00 (0.98-1.02)		.99	1.00 (0.98-1.03)		.90
**English fluency**									
	<Very good	0.70 (0.36-1.34)	.28	1.20 (0.66-2.18)		.55	1.50 (0.71-3.19)		.29
	Very good	Reference	N/A	Reference		N/A	Reference		N/A
**Has a smartphone or tablet**									
	Yes	*3.35 (2.17-5.18)*	*<.001*	*5.19 (2.83-9.51)*		*<.001*	Reference		N/A
	No	Reference	N/A	Reference		N/A	*3.35 (2.06-5.44)*		*<.001*
**Wi-Fi at home**									
	Yes	0.83 (0.48-1.43)	.50	1.85 (0.97-3.55)		.06	1.59 (0.83-3.05)		.16
	No	Reference	N/A	Reference		N/A	Reference		N/A

^a^AOR: adjusted odds ratio.

^b^N/A: not applicable.

^c^Significant relationships are italicized.

## Discussion

### Principal Findings

This study examined technology access, current use, and factors associated with preferences for health communication in a large sample of predominately low-income South Asian immigrants with prediabetes or T2D. We found a moderately high level of technology ownership and social media usage, and that participants who were of the female sex, currently employed, not married, had high school education or below, and no smart device were less likely to report interest in mHealth programs.

Our data revealed that approximately three-quarters of South Asian immigrants in NYC had a smartphone and over 60% used social media or text messaging apps. These results revealed some gaps in access to smart devices between South Asian immigrants and the general US population. According to national mobile device ownership data compiled by the Pew Research Center, 85% of the overall US general population had a smartphone, including 85% of non-Hispanic White adults, 85% of non-Hispanic Black adults, and 83% of Hispanic adults [35]. While over 50% of the US general population owns a tablet, less than 2% of the South Asian immigrants in our sample reported owning a tablet. These data suggest that underserved low-income South Asian immigrants may need additional resources and infrastructure support to benefit from telemedicine or telehealth programs in the post–COVID-19 era.

Compared to male participants, female participants were less likely to own a smartphone, have health-related apps on the smartphone, use social media apps, or report interest in text messages– or video-based diabetes information. These differences may be explained by the different sociodemographic characteristics of female and male participants in our study. Compared to male participants, female participants were more likely to have a lower level of education and to be unemployed, which have been reported to be significant predictors of technology engagement and interest in mHealth-related interventions [36-39]. In addition, the gender norms in South Asian culture may also contribute to these disparities. Past studies have noted patriarchal cultural norms in South Asian families, contributing to differences in health care–seeking and behaviors between men and women [30]. Future research studies may need to explore culturally tailored strategies that may better engage South Asian women in health and mHealth interventions [40].

Our findings also suggested that participants with less than a high school education, currently employed, and not having a smart device were more likely to report no interest in mHealth interventions or skip the question. These factors were consistent with several prior reports, which found that education, employment status, sex, and access to technologies significantly affect people’s current and future interest in mHealth interventions [36,38,40], as well as a study among South Asians in Canada [41]. Those with limited education and access to technology may have less exposure and experience with technologies and have limited digital literacy, and thus show no or little interest in technology-based interventions [38,39,42]. Future studies need to consider how to reach this group and design an appropriate intervention that meets their needs, which can help to ensure equity in the implementation of diabetes interventions and ultimately equity in health disparities driven by social factors.

### Strengths and Limitations

There are several limitations of this study to note. We used available data from two clinical trials that aimed to test the effectiveness of an integrated intervention on diabetes prevention and management in South Asian immigrants in the United States [31,32]. To minimize participant burden, we did not include an extensive list of questions about digital literacy, prior experience with various technologies, and current use of various apps or health websites. In addition, some of the data were collected during the COVID-19 pandemic. Due to remote work and learning, some participants may have purchased a smartphone or Wi-Fi or used telemedicine services due to COVID-19 [43,44]. This may lead to higher rates of smart device ownership or interest in mHealth interventions. Finally, the study sample consisted of predominately low-income South Asian immigrants, largely those from Bangladesh, who participated as volunteers in our clinical trials; therefore, our findings related to technology use may not be generalizable to the broader South Asian community in NYC.

We also want to highlight several strengths of the study. This is a relatively large sample size with a sizeable number of male participants for a health intervention. All participants were recruited from clinical settings, which greatly enhances the representation of our sample. In addition, bilingual and bicultural CHWs took the lead in recruitment, which makes the inclusion of non-English–speaking participants possible. These populations were often overlooked or not included in clinical trials that focus on English-speaking populations [45,46].

### Practice Implications

Compared to those of the general US population, the rates of technology access and adoption are relatively lower among underserved low-income South Asian immigrants, particularly among low-income South Asian women. When working with this community, it may be helpful to consider basic and simple technologies such as telephone calls and basic text messages. Although it is possible to engage this community in telemedicine or telehealth programs, this requires hands-on technology training and community engagement strategies. Due to COVID-19, our group transformed several in-person diabetes intervention programs into remote formats (eg, Zoom-based group sessions). Our preliminary data demonstrated great engagement and retention [15]. Our data also revealed that South Asian women were less likely to have access to technology or interest in mHealth interventions. Future work needs to explore barriers and develop tailored strategies to engage this unique group.

### Conclusions

We found that technology ownership, access, and social media usage were moderately high in primarily low-income South Asian immigrants living in NYC with a diagnosis of prediabetes or diabetes. Significantly higher usage and technology access was seen among male participants than among female participants. Most participants reported interest in receiving diabetes-related information via text messaging or videos. However, disparities existed within our study sample. Female participants who had limited education and no smart device were more likely to report no interest in mHealth interventions. Future studies may consider strategies to engage South Asian women and leverage text messaging and social media apps as a potential strategy to disseminate culturally and linguistically adapted diabetes information in this underserved immigrant population.

## Abbreviations

AOR: adjusted odds ratio
CHW: community health worker
HbA1c: hemoglobin A1c
IRB: institutional review board
LEP: limited English proficiency
mHealth: mobile health
NYC: New York City
T2D: type 2 diabetes

## Data Availability

The data sets generated during and/or analyzed during this study are available from the corresponding author on reasonable request.
